# Urban Scaling of Health Outcomes: a Scoping Review

**DOI:** 10.1007/s11524-021-00577-4

**Published:** 2022-05-05

**Authors:** Edwin M. McCulley, Pricila H. Mullachery, Ana F. Ortigoza, Daniel A. Rodríguez, Ana V. Diez Roux, Usama Bilal

**Affiliations:** 1 Urban Health Collaborative, Drexel University Dornsife School of Public Health, 3600 Market St, 7th floor, Philadelphia, PA 19104 USA; 2 Department of Epidemiology and Biostatistics, Drexel University Dornsife School of Public Health, Philadelphia, PA USA; 3 Department of City and Regional Planning, University of California Berkeley, Berkeley, CA USA

**Keywords:** Urban scaling, City size, City growth, Urbanization, Urban health, Complex systems

## Abstract

Urban scaling is a framework that describes how city-level characteristics scale with variations in city size. This scoping review mapped the existing evidence on the urban scaling of health outcomes to identify gaps and inform future research. Using a structured search strategy, we identified and reviewed a total of 102 studies, a majority set in high-income countries using diverse city definitions. We found several historical studies that examined the dynamic relationships between city size and mortality occurring during the nineteenth and early twentieth centuries. In more recent years, we documented heterogeneity in the relation between city size and health. Measles and influenza are influenced by city size in conjunction with other factors like geographic proximity, while STIs, HIV, and dengue tend to occur more frequently in larger cities. NCDs showed a heterogeneous pattern that depends on the specific outcome and context. Homicides and other crimes are more common in larger cities, suicides are more common in smaller cities, and traffic-related injuries show a less clear pattern that differs by context and type of injury. Future research should aim to understand the consequences of urban growth on health outcomes in low- and middle-income countries, capitalize on longitudinal designs, systematically adjust for covariates, and examine the implications of using different city definitions.

## Introduction

More than one half of the world population now lives in urban areas [1]. Cities present unique challenges for the well-being of their residents and their shared environment [2]. The United Nation’s New Urban Agenda further highlights the importance of urban health research in achieving Sustainable Development Goals such as ending poverty, hunger, and creating sustainable cities [3, 4]. In a world undergoing rapid urbanization, understanding how city-level factors change with city size can be instrumental in the creation of a unified theory of city living: a predictive framework for how urbanization and city growth affects society and the environment [5–8]. This theory would allow, among other things, for a better understanding of how health outcomes vary across the continuum of city size, and how variations in these outcomes may be associated with city-level factors and underlying policies which are important to improve planetary health.

**Fig. 1:**
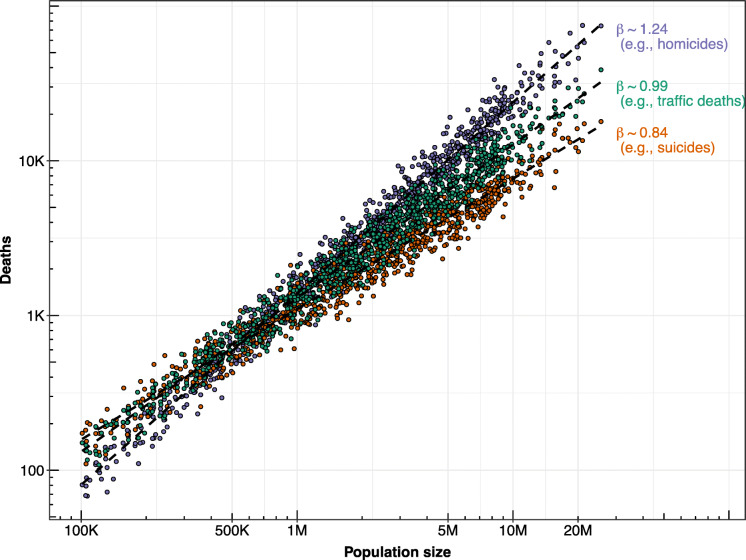
Example of three urban scaling relationships (superlinear for homicides, linear for traffic deaths, and sublinear for suicides). *Footnote:* Data simulated using scaling coefficients from Melo et al. [89] for Brazilian cities

Cities are complex systems where the dynamics of population size and social interaction give rise to emergent phenomena known as urban scaling [9]. Urban scaling describes the processes by which urban features such as economic features, wealth, crime, pollution, consumption patterns, and energy expenditure vary with changes in city size (i.e., population growth) [6]. A *linear scaling* response indicates no relationship between the urban feature and city size. For example, the amount of energy consumption per household is relatively similar across cities of similar size [5, 7]. Some characteristics of cities, for example road infrastructure, show *sublinear scaling* which means that as cities grow in size, the amount of road length and gas stations, relative to population size, decreases [5, 7]. In contrast, other features of the urban environment such as the relative amount of wealth, innovation, crime, and pollution per capita increases as cities grow in size, a phenomenon known as *superlinear scaling* [5, 7]. The way cities grow is also relevant to the scaling phenomena. While often treated as a static feature of cities, city size is the result of dynamic processes that imply many different types and rates of growth [6–8]. Figure 1 shows an example of three scaling responses for three hypothetical types of causes of death.

A large body of literature has explored urban-rural differences in health and has originated the urban penalty and urban advantage theories which posit deleterious or positive overall impacts of urban living for population health [2, 10]. However, urban-rural comparisons are often limited by the fact that cities are heterogenous in many features, including city population size. Additionally, while the urban-rural framework can provide convenient comparisons, the urban penalty and advantage theories are limited by the complexity and diversity of cities, which tend to vary across the globe; suggesting the benefits and risk of urban living are not uniform [2]. Given the complex and diverse nature of cities, there is an inherent need for a framework to outline and characterize the dynamic relationship between city characteristics and health.

Current literature applying the concept of urban scaling to health is scarce, with most research focusing on the scaling properties of factors that are determinants of health [5, 7, 11–14]. Understanding urban population dynamics, and subsequent scaling laws, are the first steps toward developing theories that describe the relationship between city characteristics and population health, with many of these characteristics being meaningful policy levers in terms of sustainability, resource limits, and healthy governance [2–4]. In this study, we review the evidence pertaining to the urban scaling of health outcomes, that is, how health outcomes scale with city size.

## Methods

The main objective of this scoping review was to map the existing evidence pertaining to the urban scaling properties of health outcomes. We followed the framework of the Joanna Briggs Institute (JBI) [15] and reported methods and results using the Preferred Reporting Items for Systematic Review and Meta-Analysis Extension for Scoping Reviews (PRISMA ScR) guidelines [16]. More details on the scoping review methodology can be found in the review protocol [17].

### Search strategy and selection criteria

Briefly, we searched for empirical or review studies that investigated city or urban size, growth, or urbanization, in relation to any health outcome, health behavior, or risk factor including prevalence, incidence, and mortality. The structured search strategy was executed in English, Spanish, and Portuguese utilizing the MEDLINE (accessed via PubMed) and Latin American & Caribbean Health Science Literature (LILACS) databases, with no time restrictions. Duplicate studies were removed, and the remaining studies were then screened for inclusion by two members of the research team (EMM and UB), regardless of study design and research quality. We excluded studies such as commentaries, studies with other primary objectives, and studies written in languages other than English, Spanish, Portuguese. Full-text studies were reviewed in duplicate by four members of the research team (EMM, UB, PHM, and AFO), with discrepancies resolved by consensus.

The key exposure of interest was any measure of city size or growth. We defined city size as a simple count of individuals residing in a city at a given point in time, and growth was defined as a change in the number of individuals residing in a city over time. Although these two exposures are similar, differentiating between the two is critical in understanding any relationship between exposure(s) and outcome(s). Health outcomes were categorized according to the World Health Organization classification system for diseases and injuries [18] into: communicable, maternal, neonatal and nutritional conditions (CMNN), non-communicable diseases (NCDs) and their risk factors, and external causes or injuries. To determine which studies utilized an urban scaling framework, we identified scaling studies as those that specifically and explicitly presented findings in terms of an urban scaling response (i.e., sublinear, linear & superlinear scaling).

### Presentation of results

We presented results by study inclusion/exclusion, study design, and methods, followed by key findings pertaining to the urban scaling of health outcomes for scaling and non-scaling studies in each category of health outcomes. We also summarized adjustment for covariates in scaling studies.

### Role of the funding source

The funding sources had no role in study design, data collection, analysis, interpretation of data, writing, or in the decision to submit the manuscript. All authors had full access to all the data in the study and accept responsibility for the decision to submit the manuscript for publication.

## Results

### Study inclusion/exclusion

**Fig. 2:**
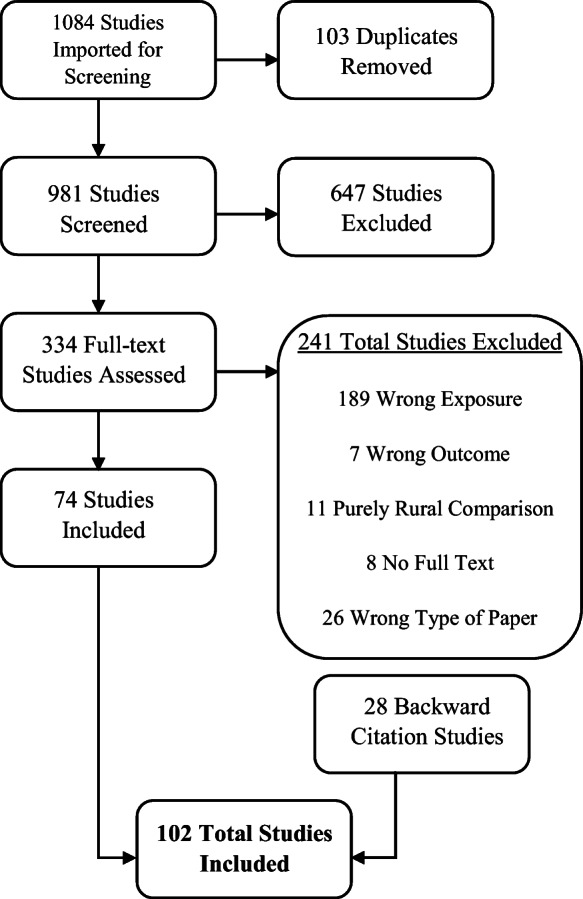
PRISMA flowchart. *Footnote:* Wrong exposure refers to studies that do not have a city size or growth exposure. Wrong outcome refers to studies not using a health outcome. Wrong type of paper refers to editorials or opinion pieces. Purely rural comparison refers to studies that do not compare cities, but only urban and rural areas. Wrong setting refers to studies that were not set in cities

The PRISMA flowchart (Fig. 2) depicts the results of our review process. Our search yielded a total of 1084 studies. After title/abstract review, we found 334 studies eligible for full-text review, of which 74 were finally included. The most common reasons for exclusion were no exposure measure (e.g., city size or growth), commentaries, purely urban to rural comparisons (no comparison between cities), and no clearly defined health outcome. In addition to the 74 studies identified from the initial search, 28 additional studies were included through backward search of citations (cited by an included study), resulting in a total of 102 studies published from 1946 to 2019. A majority of the evidence was published in English (n = 98), and nearly 60% was published between 2010 and 2019. Only 15% of studies employed a scaling framework in their analyses (n = 15).

### Study design and methods

Tables 1 and 2 describe overall characteristics of each non-scaling and scaling study, respectively. Ecological studies were the most common study design (n = 79), followed by individual level studies (n = 6), systematic reviews (n = 4), and simulation studies (n = 13). Around 90% of the studies used cross-sectional analyses (n = 93), and 9% used longitudinal analyses (n = 9). Roughly 73% of the studies were set in high-income countries (n = 75), and 17% in low- and middle-income countries (n = 17). A majority of the results were set in the Americas (n = 56), primarily in the USA (n = 42), and Brazil (n = 8), while the rest were set in Europe (n = 22) or Asia (n = 8). Additionally, 12 studies examined the urban scaling of health outcomes in numerous cities across more than one country.

**Table 1:** Characteristics of Non-Scaling Manuscripts (n=84)

Characteristic		CMNN*		NCD*		EXTERNAL CAUSES/ INJURIES*		ALL-CAUSE MORTALITY*		OTHER*	
		N	Ref.	N	Ref.	N	Ref.	N	Ref.	N	Ref.
Exposure	Population Size	33	[21–23, 36-42, 44, 45, 50-57, 59-70, 116]	18	[23, 25, 41-43, 73, 74, 76, 85, 86, 88, 116-122]	4	[41, 42, 97, 123]	4	[19, 20, 24, 26]	1	[96]
	Population Size & Relative Location	5	[35, 46-49]	10	[72, 75, 77-80, 82-84, 124]	4	[94, 95, 98, 99]	0		1	[81]
	Population Growth	3	[33, 58, 103]	0		0		3	[27, 125, 126]	1	[127]
	Other	2	[34, 128]	4	[87, 128-130]	1	[128]	0		0	
Outcome Measure	Mortality Rates	14	[21, 23, 40, 41, 44, 46-52, 54, 57]	10	[23, 25, 41, 74, 75, 82-84, 120, 124]	3	[41, 98, 99]	6	[20, 24, 26, 27, 126, 127]	0	
	Prevalence	2	[35, 37]	11	[78-80, 85–88, 118, 120, 121, 130]	3	[94, 97, 122]	0		3	[81, 96, 128]
	Incidence	16	[22, 33, 36, 38, 39, 42, 45, 55, 58, 60, 61, 66, 67, 69, 70, 103]	5	[42, 72, 76, 116, 122]	1	[42]	0		0	
	Several	1	[128]	6	[43, 73, 77, 117, 128, 130]	2	[95, 128]	1	[19]	0	
	Other	10	[34, 53, 56, 59, 62-65, 68, 115]	0		0		0		0	
City Definition	Administrative Unit	19	[21–23, 33, 34, 38-42, 44, 50, 51, 56, 58, 59, 64, 69, 103]	16	[23, 25, 41-43, 72, 74, 76, 77, 85, 86, 88, 118, 120, 122, 130]	4	[41, 42, 98, 123]	5	[19, 20, 27, 125, 126]	1	[127]
	Official Metropolitan Area	7	[35–37, 46–49]	11	[75, 78-80, 82-84, 116, 117, 119, 124]	4	[94, 95, 97, 99]	1	[26]	2	[81, 96]
	Other	16	[45, 52–55, 57, 60–63, 65–68, 70, 115]	4	[73, 87, 121, 129]	0		0		0	
	Unclear	1	[128]	1	[128]	1	[128]	1	[24]	0	
Setting-Time*	2^nd^ Half of 19^th^ Century	2	[23, 103]	1	[23]	0		4	[19, 20, 24, 27]	0	
	1^st^ Half of 20^th^ Century	19	[21–23, 40, 41, 50–54, 60, 62, 63, 65, 66, 68–70, 103]	2	[23, 41]	1	[41]	3	[19, 26, 27]	0	
	2^nd^ Half of 20^th^ Century	20	[33, 34, 37–39, 44, 46, 57, 60–62, 64–70, 114, 128]	18	[25, 72–75, 82–85, 87, 88, 116, 118, 119, 120, 128–130]	6	[94, 95, 97, 99, 123, 128]	5	[20, 26, 27, 125, 126]	1	[127]
	21^st^ Century	20	[33–36, 38, 39, 42, 44, 45, 47–49, 55-59, 64, 114, 127]	16	[42, 43, 76–80, 83, 86, 87, 117, 120, 121, 124, 128, 130]	6	[42, 97-99, 123, 128]	2	[27, 125]	2	[81, 96]
Setting-Location	Americas	21	[21–23, 34, 35, 39–42, 44, 46–49, 51, 53, 55–57, 59, 63]	18	[23, 25, 41, 42, 72, 74, 75, 78–80, 82–84, 86, 117, 119, 124, 130]	7	[41, 42, 94, 95, 97-99]	1	[26]	1	[81]
	Africa	3	[45, 64, 103]	0		0		0		1	[128]
	Europe	11	[37, 50, 58, 60-62, 65-69]	6	[73, 88, 116, 118, 120, 122]	1	[123]	3	[19, 24, 125]	1	[96]
	Asia	3	[33, 38, 115]	4	[43, 76, 77, 85]	0		1	[126]	0	
	Other	5	[36, 52, 54, 70, 128]	4	[87, 121, 128, 129]	1	[128]	2	[20, 27]	0	
Design-Type	Ecological	28	[21–23, 33–35, 37-42, 44-52, 54, 60, 62, 63, 67, 70, 103]	25	[23, 25, 41–43, 72, 74–80, 82–86, 88, 116, 118, 119, 122, 124, 130]	8	[41, 42, 94, 95, 97-99, 123]	7	[19, 20, 24, 26, 27, 125, 126]	1	[96]
	Experimental	13	[36, 53, 55-59, 61, 64-66, 68, 69]	0		0		0		0	
	Individual Level	1	[115]	3	[73, 117, 120]	0		0		2	[81, 128]
	Review	1	[127]	4	[87, 121, 128, 129]	1	[129]	0		0	
Design-Time	Cross-Sectional	40	[21-23, 34-37, 39-42, 44-70, 115, 128]	29	[23, 25, 41-43, 72, 74-80, 82, 85-88, 116-122, 124, 128-130]	9	[41, 42, 94, 95, 97-99, 124, 129]	4	[19, 20, 26, 127]	3	[81, 96, 128]
	Longitudinal	3	[33, 38, 103]	3	[73, 83, 84]	0		3	[24, 27, 126]	0

**Table 2:** Characteristics of Scaling Manuscripts (n=15)

Characteristic		CMNN*		NCD*		EXTERNAL CAUSES/ INJURIES*		OTHER*	
		N	Ref.	N	Ref.	N	Ref.	N	Ref.
Exposure	Population Size	4	[7, 28, 31, 32]	1	[28]	9	[7, 12, 13, 28, 89–93]	1	[28]
	Population Size & Relative Location	2	[29, 30]	1	[71]	0		1	[131]
Outcome Measure	Mortality Rates	0		1	[71]	7	[12, 13, 89–93]	0	
	Prevalence	0		0		0		1	[131]
	Incidence	5	[7, 29–32]	0		1	[7]	0	
	Several	1	[28]	1	[28]	1	[28]	1	[28]
City Definition	Administrative Unit	2	[28, 32]	2	[28, 71]	3	[28, 90, 91]	1	[28]
	Official Metropolitan Area	3	[29–31]	0		2	[12, 92]	1	[131]
	Administrative Unit & Official Metropolitan Area	1	[7]	0		4	[7, 13, 89, 93]	0	
Setting-Time*	2^nd^ Half of 20^th^ Century	3	[7, 28, 32]	2	[28, 71]	6	[7, 12, 28, 89, 91, 93]	2	[28, 131]
	21^st^ Century	6	[7, 28–32]	2	[28, 71]	8	[7, 12, 13, 28, 89, 90, 92, 93]	2	[28, 131]
Setting-Location	Americas	4	[29–32]	1	[71]	7	[12, 13, 89–93]	1	[131]
	Other	2	[7, 28]	1	[28]	2	[7, 28]	1	[28]
Design-Type	Ecological	6	[7, 28–32]	2	[28, 71]	9	[7, 12, 13, 28, 89–93]	2	[28, 131]
Design-Time	Cross-Sectional	6	[7, 28–32]	2	[28, 71]	9	[7, 12, 13, 28, 89–93]	2	[28, 131]

*Note: Citations belonging to more than 1 subcategory are listed multiple times across every applicable subcategory

The earliest studies were set in the nineteenth century in Scotland [19] and England [20], and the nineteenth and early twentieth century in the USA [21–23], while the majority were set in the twenty-first century (n = 56). The most commonly used city definitions were administrative units (n = 45) (e.g., counties, municipalities), followed by country-defined official metropolitan areas (n = 31), and other researcher-defined delineations (n = 21) that were based on satellite imagery data, relational classifications (e.g., core vs. fringe urban area), and arbitrarily assigned population size cut-points. Two studies did not present a clearly identifiable city definition, and three used several definitions concurrently.

The most common exposure among included studies was population size (n = 67), in which a simple count of the population living in the city was used, either as a continuous or categorical predictor. Other exposures included categorical predictors intended to capture levels of urbanicity (n = 23), population growth (n = 7), and study-specific measures of urbanization (n = 5). In all cases, these measures included at least one metric of city size, resulting in 95 studies using an exposure directly or indirectly based on city size, and only 7 studies using a population growth as the exposure. The most frequent class of health outcomes were, CMNN conditions (n = 49), followed by NCDs or their risk factors (n = 34), and injuries (n = 18). A few studies examined all-cause mortality (n = 7) and others had outcomes based on behaviors or health related perceptions (n = 5).

### Historical studies examining the urban penalty in high-income countries

We found a number of historical studies examining the urban penalty in the nineteenth or first half of the twentieth century in the UK and the USA, positing that urban living had adverse health impacts as a result of the unhealthy environments created by population concentration and industrialization [10]. The studies focused on the nineteenth century showed lower life expectancy in larger cities [19, 24]. Results from the early twentieth century in the USA were complex, with higher mortality in smaller cities immediately following the 1918 influenza pandemic, followed by a change in the burden of mortality from infectious disease mortality to NCD mortality in larger cities [23]. By the middle of the twentieth century NCD rates in larger cities began to stabilize and decrease over time [25], while mortality remained highest in metropolitan areas with populations greater than 50,000, except for accidents and suicides [26]. Worldwide, studies focused on the early twentieth century described rapid post-war population growth in cities linked to low urban wages and the rise of poor mega-cities [27].

### Communicable, maternal, neonatal, nutritional conditions and infant mortality

We found 49 studies that examined the association between city size or growth and rates of CMNN conditions. Six of these specifically employed a scaling framework (Table 2). In general, for cities in the USA, Brazil, and Sweden, the incidence of human immunodeficiency virus (HIV), influenza, meningitis, dengue fever, leprosy, and hepatitis A, B, and C scaled superlinearly with city size [28]. This superlinear scaling behavior was also observed for the incidence of sexually transmitted infections (STIs), specifically chlamydia, syphilis, and gonorrhea [28–31], indicating that infections of this type are more common in large cities. Two studies examined the incidence of acquired immunodeficiency syndrome (AIDS) as a function of population size, finding a superlinear behavior [7, 32]. However, a few diseases (hantavirus and leprosy) were more common in medium-sized cities [33, 34]. There was only one study looking at infant and child mortality in US and Brazilian cities, which found higher rates of infant and child mortality in small cities [28]. Overall, these studies did not adjust for covariates, except those focused on STIs, which explored the role of several city-level covariates (age distribution, racial/ethnic composition, income, education) in the generation of scaling patterns [29, 30].

We found 43 studies examining the relationship between CMNN conditions and city size without a scaling framework, most of them finding higher rates in larger cities (Table 3). In Europe and the USA, larger cities had a higher prevalence of HIV and AIDS cases [35, 36], and other STIs [37]. The incidence of vector-borne diseases such as dengue fever in Singapore and leishmaniasis in Brazil was found to be higher in larger cities compared to smaller cities [38, 39]. Additionally, mortality from tuberculosis in the USA was higher in larger cities during most of the twentieth century [40, 41]. A few diseases followed inverted u-shapes with population size (more common in medium-sized cities), including the incidence of hantavirus in China [33], or leprosy in Brazil [34]. Finally, hospitalizations due to communicable disease in Brazil and South Korea were lower in large cities [42, 43]. Aside from communicable diseases, there were several non-scaling studies of infant mortality, maternal, and neonatal conditions (n = 10), however, these findings are heterogenous and appear to vary by health outcome and geographic context. In Mexico, under 5 mortality due to birth defects was more prevalent in larger cities [44], while under 5 mortality rates were higher in smaller cities of Sub-Saharan Africa [45]. In the USA perinatal [46], infant [46–48], and child mortality rates were higher in smaller cities [49].

**Table 3:** Scaling Relationships

Classification	Scaling Relationship	Health Outcome	Setting- Location	Setting- Time	Citation(s)	Year
Communicable, Maternal, Neonatal, and Nutritional Conditions (CMNN)	Linear (No Relationship with City Size)	Hepatitis B	Brazil	2007	[28]*	2015
		Influenza	Brazil	2010		
	Sublinear (More Common in Small Cities)	Dengue	Brazil	2001		
		Infant & Child Mortality	Brazil	2012		
		Leprosy	Brazil	2001, 2002		
		Infant & Child Mortality	United States	2000-2009		
	Superlinear (More Common in Large Cities)	Infant & Child Mortality	Brazil	1981		
		Influenza	Brazil	2009		
		Hepatitis B	Brazil	2012		
		Dengue	Brazil	2012		
		AIDS cases	Brazil	1980-2012		
		HIV	Brazil	1990, 2012		
		Meningitis	Brazil	2001, 2012		
		Hepatitis A	Brazil	2007, 2012		
		Hepatitis C	Brazil	2007, 2012		
		Chlamydia	United States	2011		
		HIV	United States	2000-2009		
		Chlamydia Gonorrhea Syphilis	United States	2007-2011	[29]	2018
		Chlamydia Gonorrhea Syphilis	United States	2007-2011	[30]	2015
		Chlamydia Syphilis	United States	2007-2011	[31]	2018
		AIDS cases	United States, China, Germany	1990-2003	[7]*	2007
Non-Communicable Diseases (NCD)	Linear	Cerebrovascular Accident Mortality	Brazil	2012	[28]*	2015
		Colon Cancer Mortality	Brazil	2012		
	Sublinear	Colon Cancer Mortality	Brazil	1981		
		Diabetes Mortality	Brazil	2012		
		Diabetes Mortality	Sweden	2008-2012		
		Heart Attack Mortality	Sweden	2008-2012		
		Lung Cancer Mortality	Sweden	2008-2012		
		Chronic Respiratory Insufficiency Mortality	Sweden	2008-2012		
		Obesity	Sweden	2010-2013		
		Obesity	United States	2010		
	Superlinear	Diabetes Mortality	Brazil	1996		
		Cerebrovascular Accident Mortality	Brazil	1996		
		Heart Attack Mortality	Brazil	1981, 2012		
		Lung Cancer Mortality	Brazil	1981, 2012		
		Chronic Respiratory Insufficiency Mortality	Brazil	1981, 2012		
		Cancer Cardiac Disease Respiratory Disease Endocrine Metabolic Disease	United States	1999-2010	[71]	2018
External Causes/Injuries	Linear	Pedestrian Mortality	United States	1994-2011	[93]*	2016
		Traffic Accident Mortality	United States & Brazil	2003-2007	[89]*	2014
	Sublinear	Rape	Brazil	2009	[28]*	2015
		Traffic Accident Mortality	Brazil	2012		
		Suicide	Brazil	1981, 1995		
		Suicide	Brazil	2005-2014	[90]*	2018
		Suicide	Sweden	2008-2012	[28]*	2015
		Drug Poisoning	United States	2000		
		Suicide	United States & Brazil	1992-2009	[89]*	2014
	Superlinear	Traffic Accident Mortality	Brazil	1981	[28]*	2015
		Homicide Mortality	Brazil	2000	[91]	2013
		Homicide Mortality	Brazil	2010	[13]	2014
		Rape	Brazil	2012	[28]*	2015
		Homicide, Traffic Accident Mortality	Brazil	2005-2014	[90]*	2018
		Homicide Mortality	Several	2003-2009	[92]	2012
		Rape	Sweden	2013	[28]*	2015
		Homicide Mortality	United States	1969-2006	[12]	2010
		Non-Pedestrian Mortality	United States	1994-2011	[93]*	2016
		Excessive Alcohol Consumption	United States	2006-2012	[28]*	2015
		Violent Crimes	United States	2009-2011		
		Homicide Mortality	United States & Brazil	1992-2009	[89]*	2014
		Homicide Mortality	United States, China, Germany	1990-2003	[7]*	2007
Other	Linear	Organ Donation	United States	1995-2008	[131]	2011
	Sublinear	Physical Inactivity	United States	2010	[28]*	2015

*Note: Citations with health outcomes belonging to multiple classifications are listed multiple times across applicable classifications

Two epidemic diseases have frequently been linked to population size: influenza and measles. We found a total of 11 studies that examined the relationship between influenza and city size, one using a scaling framework [21]. Six of these examined the 1918 influenza pandemic, finding that while mortality was generally higher in urban areas as compared to rural areas [50], there was either a weak correlation with city size [51–53], or slightly higher mortality in smaller cities [21, 50, 54]. These results were consistent with the five studies examining seasonal influenza, finding that geographic location matters more than city size [55–57], although population growth [58] and size [59] may play a role in shaping seasonal flu epidemics.

We found a total of 11 studies examining the relationship between measles and city size, two of them using a scaling framework [60, 61]. All measles studies characterized how city size affected the shape of epidemics, including the intensity and frequency of fadeouts. This started with the works of Bartlett [62, 63], who characterized a critical community size (CCS) threshold of 300–400,000 persons, above which cities do not experience fadeouts in measles incidence, the temporary disappearance of measles from a population. This CCS threshold is influenced by birth rates and, nowadays, by vaccination coverage [64, 65]. Several studies suggested that in populations below the critical size, the probability of fadeouts increase as population size decreases [60, 62–70]. A second critical aspect of the measles dynamics is the presence of a spatial hierarchy, where epidemics of measles move from larger “donor” cities to nearby smaller “recipient” towns [69, 70], this phenomenon scales superlinearly with donor city size, so that larger cities are more likely to be the source of regional epidemics [61]. Last, the incidence of pertussis, another frequent but vaccine-preventable childhood disease, follows a pattern similar to measles [22].

### Non-communicable diseases

Of the 34 NCD studies identified in the review, there were only 2 scaling studies (Table 3). In a study of four major classes of NCDs in large urban US counties, the authors found a superlinear scaling behavior for deaths due to cancer, circulatory, respiratory, endocrine, nutritional and metabolic diseases [71]. However, the authors found that this superlinear behavior was sensitive to the size of included counties, as the relationships turned sublinear when only the largest countries were included, possibly indicating higher mortality in mid-sized cities. In a study with multiple outcomes in US, Brazilian and Swedish cities [28], the NCD results varied by context. For example, heart attack mortality, lung cancer, and respiratory insufficiency, scaled superlinearly in Brazil and sublinearly in Sweden [28]. Additionally, in the USA and Sweden, obesity scaled sublinearly. This same study suggested that physical inactivity scaled sublinearly, and excessive alcohol consumption scaled superlinearly in US cities [28]. Only one of these studies explored the effects of adjustment for covariates by including covariates of income and population density [71].

We found 32 non-scaling studies that examined the relationship between city size or growth and NCDs. The association between city size and cancer varied by type and location. The incidence of acute lymphocytic leukemia was higher in large US cities [72], while in Europe and the USA lung cancer and its major risk factor, smoking, were more common in larger than in smaller cities in the second half of the twentieth century [73, 74], a pattern consistent with higher mortality by other cancer types with increasing urbanization levels [75]. In South Korea thyroid and colorectal cancers were more common in larger cities, but gastric and lung cancers more common in smaller cities [76]. The prevalence of cardio-metabolic conditions varied by city size and location. Larger cities in China had a higher prevalence of obesity [77], while in the USA the prevalence of obesity was lower in large cities [78–80], a result consistent with higher rates of physical inactivity in less urbanized areas [81]. Several findings indicate that coronary heart disease mortality in the USA used to be more prevalent in larger cities, compared to their smaller counterparts [41, 82–84]. Last, the prevalence of psychiatric disorders such as clinical depression and anxiety disorder increased with city size [85–88].

### External causes/injuries

Health outcomes classified as external causes and injuries are among the health outcomes more frequently studied from a scaling perspective (n = 9, Table 3). Overall, these findings largely suggested that homicides scale superlinearly with city size [89–92], but a study in Brazil suggested that this result may not be linear, with potential for mid-sized cities to have higher homicide rates [13]. Aside from homicides, one study found that other violent crimes such as rape and domestic physical violence scaled superlinearly [28]. Suicide mortality in US and Brazilian cities scaled sublinearly [89, 90]. Studies on traffic-related injuries displayed linear [89], superlinear [28, 90], and sublinear behaviors [28]. These differences may be related to the type of traffic-related mortality, as a study in US cities found that pedestrian fatalities scaled sublinearly with population size, and non-pedestrian fatalities displayed a superlinear scaling response [93]. For the most part, these scaling studies did not adjust for any covariates; except for two studies, which adjusted for educational attainment [31] and income per capita [93], respectively.

We found 9 non-scaling studies of injuries. Among these non-scaling studies, homicide was more common in larger cities compared to smaller cities [41, 94, 95]. Levels of perceived insecurity were also found to be higher in larger cities than in smaller cities [96]. A few studies suggested that other injuries, such as those from motor vehicle accidents and suicide are more common in less populated areas [97, 98]. Out-of-hospital injury related mortality rates were higher in less urbanized areas [43], while injury hospitalization rates in Brazil were highest in mid-sized cities [42]. Last, in a small study using data from 18 cities in New Mexico, USA, the rate of unintentional drug overdoses was higher in larger cities than in smaller cities [99].

## Discussion

In this scoping review, we mapped evidence regarding the associations between city size or growth and health outcomes, with a focus on studies with an explicit scaling framework. We highlight five key findings. First, we found a diverse literature from many different geographical and temporal settings and outcomes, that included heterogeneous city definitions and different operationalizations of city size (e.g., continuous, as is the case for all scaling studies and some non-scaling studies, as well as categorical). Second, we found evidence of an urban penalty with higher mortality and worse health outcomes in larger cities of high-income countries, at least during the nineteenth century, that shifted in the early twentieth century toward lower mortality in larger cities. Third, we found that two key diseases with an epidemic component, measles, and influenza, are influenced by city size in conjunction with other factors like geographic proximity and transmission potential, while other communicable diseases such as STIs, HIV, and dengue tend to occur more frequently in larger cities. Fourth, we found that NCDs show a heterogeneous pattern that depends on the specific outcome and context. Fifth, homicides and other crimes are more common in larger cities, suicides are more common in smaller cities, and traffic-related injuries show a less clear pattern that may differ by context and type of injury.

A majority of the studies in this review were set in high-income countries (75 out of 102, 74%). While we captured a few studies from low- and middle-income countries (LMIC), such as Brazil and Mexico, the absence of evidence examining the urban scaling in other settings is a clear gap in the literature. This lack of evidence is especially worrisome for low-income countries, where poor sanitation, inequalities in resource availability, and overcrowding are especially prevalent in urban areas and may have a large influence on scaling patterns [100]. Furthermore, most future urban population growth is expected to occur in LMICs, specifically in Latin America, Asia, and Africa, and understanding the consequences of urban growth in these settings is key to achieving the Sustainable Development Goals [101] and should be a priority of future research.

One key aspect of being able to compare cities is having a clear definition of their boundaries [102]. In this scoping review, we found large heterogeneity in the way cities are defined. There is no single universally accepted definition of a city, and more often than not, the way cities are defined varies across countries and regions. While administrative units were the most used city definition, their primary purpose is administrative, and they may not represent actual city boundaries. Understanding the consequences of different city definitions on the scaling properties of health outcomes is a key direction of future research, as previous studies have highlighted that the scaling laws of some city features may vary systematically by city definition [11]. In a small number of studies we were not able to even identify what the authors referred to as “city”, which creates issues for reproducibility. Future research on urban health should clearly define what is meant by “city” and how boundaries are defined.

Our second key finding is that an urban penalty was present in the nineteenth and early twentieth century for studies set in what are now high-income countries [19, 22–27, 103], with a shift occurring during the first half of the twentieth century toward lower mortality, especially due to communicable diseases, in larger cities of high-income countries [23]. The shift in mortality is likely attributable to changes in both rural and urban areas [2]. However, the heterogeneity in outcomes observed for cities of similar size in most scaling studies points to other city characteristics that are driving health. The emergence of these characteristics depends not only on size, but also on differences in geographic context, connectivity, resource availability, and economic growth, among many other factors [2]. Aside from being complex, city populations are among the most diverse; and while urbanization can affect health, these effects are heterogeneous for different populations, resulting in inequities at multiple levels [104]. Additionally, the observed shift in mortality may be related to changes in the urbanization processes [2, 27], evident in present day LMICs where rapid urbanization and development may contribute to unsafe settlement conditions and poor access to services, which can further exacerbate the urban penalty [105]. Whether the shifts in disease burden that originally occurred in cities of high-income countries are being replicated currently in LMIC cities has yet to be studied, precluding a complete understanding of this phenomenon, so future studies should leverage cross-national comparisons of cities to understand the dynamic associations between urbanization and health in countries at different stages of development [106].

Our third finding identified complex associations of city size and growth with certain diseases such as measles and influenza, and superlinear associations with city size for other commonly studied communicable diseases such as STIs, HIV, and dengue fever. A number of studies examined the 1918 influenza pandemic, with mixed evidence regarding the role of city size, consistent with studies on seasonal influenza. On the other hand, for measles, city size has a clear effect on the size and shape of epidemic waves [69, 70], as factors such as the critical community size, spatial hierarchies, and fadeout probabilities are all related to city size [68]. Last, STIs follow a consistent superlinear scaling pattern [30], but the scaling behavior of specific STIs is heterogenous and may depend on variability in disease transmission [29]. The effect of transmission variability on disease dynamics has been reported before [107, 108], and currently represents a potential avenue of future research in understanding the dynamics of large outbreaks such as the COVID-19 pandemic [109, 110].

Our fourth finding was that NCDs show a heterogenous pattern which varies based on health outcome, geographic context, developmental stage, and other factors. This is evident in the findings that cardiometabolic conditions scaled differentially in cities of the USA, Brazil, and China, where the USA tends to display a sublinear behavior (outcomes more common in smaller cities) while other countries display superlinear behaviors (outcomes more common in larger cities). While NCDs were the second most common class of health outcome in the review, there is limited evidence about the urban scaling of NCDs to date.

Our fifth key finding, is that the scaling properties of injuries were mostly consistent, indicating that homicides and other serious crimes were more common in larger cities, and suicides were more common in smaller cities. However, the scaling properties of road-traffic injuries were less clear and seemed to vary by type of injury (e.g., pedestrian vs. other road users) [93]. This may also be due to underestimation resulting in relatively low counts of injuries, compared to broader causes of death, which may lead to statistical issues in estimating scaling coefficients when a number of cities have zero counts of a specific injury [92].

Our review identified a few directions for future research on the urban scaling of health outcomes. We found very little research examining population growth as an exposure. The study of population growth in longitudinal study designs allows better inferences regarding the possible causal link between city size and health than cross-sectional analyses of city size. Drawing inferences regarding the links between city size and city outcomes from cross-sectional analyses of city size relies on an important assumption: the absence of confounding by other factors associated with city size (i.e., differences between cities of different sizes are equivalent to differences associated with changes in city size for a given city over time, which holds at least time invariant city factors constant). This is also known as the assumption of ergodicity (i.e., lack of path dependence), or no impact of how the city arrived to that population number [111]. Recent studies have challenged this assumption, finding that the longitudinal scaling properties of urban features may differ from the cross-sectional properties [112–114]. Better understanding of the links between city size and health requires longitudinal analyses that examine population growth within cities over time as well as attention to the type of city growth and the processes driving growth.

While scaling studies aim to describe changes in city outcomes with changes in city size, the scaling framework also allows for the differentiation between size-related and place-specific effects, as proposed by Bettencourt, Lobo, Strumsky & West [12]. This is achieved through the mapping and examination of regression residuals from the basic scaling equation, which contain deviations from the empirically estimated scaling power law. These residuals are dimensionless indicators, independent of city size, that can provide quantitative information about the performance of urban areas and allow for calculation of correlations with other city-level predictors. These other city-level predictors include city-level policies, social environment features (e.g., levels of poverty, inequality, segregation, etc.), and physical and built environment characteristics (e.g. climate, air pollution, urban landscape, street design, etc.), among others. The key contribution of a scaling analysis that includes an exploration of residuals would be the joint interpretation of both size-related patterns (e.g., a scaling coefficient above 1 indicating a higher homicide rate in larger cities) and city-specific effects derived from other city features independent of city size (e.g., cities with higher income inequality having a higher homicide rate).

Finally, all studies included in this review have a common objective of examining the relationship between city size and some health outcome(s); features characteristic of the urban scaling framework. However, we found heterogeneity in how these studies were conducted, in terms of definitions, operationalizations of city size, and the presence of (or lack thereof) adjustment variables. For example, we only found a few studies that examined how introducing adjustment variables changed scaling patterns [29–31, 39, 71, 93]. Future research should be transparent about the inclusion of relevant covariates, as adequately controlling for these covariates can influence the scaling response and also provide meaningful evidence on the relationship between covariates (e.g., income, education, population density) and health in cities. For example, given the important role of age in driving mortality, studies of the scaling of deaths with city size should consider how adjusting for age may change scaling coefficients.

We acknowledge some limitations. First, our search strategy may have missed some studies on city size/growth and health, especially if they were published in journals not indexed in the databases we searched. This may be especially important for studies published in the early twentieth century, which may not be entirely captured in these databases. However, in order to increase the scope of the review, we also used a backwards search to identify references cited by included studies. Second, we did not complete a forward search (i.e., a search for papers citing included studies). Consequently, we may have missed studies relevant to our objectives. Additionally, the decision to search only two databases (PubMed and LILACS) may have excluded relevant studies. Last, given the broad scope of our review, we could not present the results of each study in detail. However, as is the goal of scoping reviews, our main objective was to map the available evidence and identify gaps for future research. We have also provided in Appendix Table 4 the full scope of our review, detailing all reviewed studies. We also acknowledge several strengths. This scoping review has provided an initial comprehensive map of evidence on the urban scaling of health outcomes. We reviewed 102 studies in total, drawing attention to several factors that may contribute to inconsistencies between studies, including exposure, and city definitions. The scoping review was not limited to a single language and was able to capture evidence in English, Spanish, and Portuguese.

## Conclusions

In this scoping review, we have identified a rich and complex evidence landscape on the urban scaling of health outcomes and the relationship between city size and health. However, we have identified several gaps that merit future research, including a paucity of research in LMICs urban areas and across a variety of countries in different settings, along with a lack of clarity and consistency in city definitions, and how different definitions may lead to changes in inferences. We also identified several aspects where current research in scaling may help in understanding disease dynamics, including the exploration of the complexity of transmission of epidemic diseases, the recognition of the importance of studying population growth (i.e., longitudinal population size), the use of deviations from the scaling law to study predictors of health outcomes, and greater transparency about decisions regarding adjustment for important covariates. With growing urban populations worldwide, the continuous challenge of non-communicable diseases, the importance of injury mortality in premature mortality, and the (re-)emergence of infectious diseases, understanding the consequences of our urban world seems key in the design and planning of interventions to address unmet public health needs.

## Appendix

**Table 4:** Characteristics of scoping review evidence

Citation	Language	Objectives	Study Design	Study Design Time	Study Population	Study Setting Time	Study Setting Location
[35]	English	To assess the role of poverty in racial/ethnic disparities in HIV prevalence across counties with different levels of urbanization	Ecological	Cross-Sectional	Black, White, and Hispanic persons aged 13 and older diagnosed with HIV between 2007-2009	2009	USA
[25]	English	To examine how CHD mortality varies among rural residents exposed to different degrees of urban influence	Ecological	Cross-Sectional	White males of North Carolina aged 55 to 64	1951-1953, 1959-1961	USA
[73]	English	To assess inequalities and trends in smoking prevalence between urban and non-urban residents	Individual-Level	Longitudinal	Adult residents of 6 European countries ages 25-79	1985-2000	Sweden, Denmark, Finland, Germany, Italy, Spain
[81]	English	To estimate levels of non-occupational leisure time physical activity by degree of urbanization and region	Individual-Level	Cross-Sectional	Adult US residents	2001	USA
Ponte, E. V., Cruz, A. A., Athanazio, R., Carvalho-Pinto, R., Fernandes, F. L., Barreto, M. L., & Stelmach, R. (2018). Urbanization is associated with increased asthma morbidity and mortality in Brazil. The clinical respiratory journal, 12(2), 410-417.	English	To measure the association between level of urbanization and asthma burden	Ecological	Cross-Sectional	Residents ages 5-29 diagnosed with asthma	1999-2001, 2009-2011	Brazil
Ogundipe, F., Kodadhala, V., Ogundipe, T., Mehari, A., & Gillum, R. (2019). Disparities in Sepsis Mortality by Region, Urbanization, and Race in the USA: a Multiple Cause of Death Analysis. Journal of racial and ethnic health disparities, 6(3), 546-551.	English	To assess disparities in sepsis mortality by urbanization, region, and race in the US	Ecological	Cross-Sectional	US residents aged 15 and older	2013-2016	USA
[96]	English	To examine the association between urbanization and perceived levels of insecurity in patients with mood or anxiety disorder	Ecological	Cross-Sectional	Adults ages 18-65 with a diagnosis of anxiety or unipolar affective disorder	2011	Italy
[74]	English	To describe geographical patterns in lung cancer mortality in US counties by level of urbanization	Ecological	Cross-Sectional	US residents with lung cancer cause of death	1970-1979, 1980-1987	USA
[85]	English	To examine the association between the incidence of hysteria and urbanization	Ecological	Cross-Sectional	Females with a diagnosis of hysteria or depression	1952-1956, 1957-1973	Japan
[103]	English	To establish a likely time period for cross-species contamination with HIV and explore how risk factors such as GUD incidence, city growth, and gender distributions varied in relevant regions in Africa.	Ecological	Longitudinal	Population of 12 cities in Central and West Africa. Simulation was done with single men and women and sex workers	~1880-1940	Central and West Africa
[72]	English	To examine whether the incidence of acute lymphocytic leukemia increases with increasing levels of urbanization	Ecological	Cross-Sectional	White US children ages 0-4	1995-2000	USA
[44]	Spanish	To identify spatial relationships between birth defect mortality and socio-demographic characteristics of urbanization in cities with higher levels of under-five mortality rate	Ecological	Cross-Sectional	Children aged 0-4 in Mexican municipalities	1998-2006	Mexico
[42]	Portuguese	To study the association between socioeconomic determinants and hospitalizations due to primary care sensitive conditions	Ecological	Cross-Sectional	Everyone living in the state of Espiritu Santo, Brazil	2010	78 municipalities in a state of Brazil
Levine, R. V., Lynch, K., Miyake, K., & Lucia, M. (1989). The Type A city: Coronary heart disease and the pace of life. Journal of Behavioral Medicine, 12(6), 509-524.	English	To examine the relationship between pace of life and CHD in US metropolitan areas of different sizes	Ecological	Cross-Sectional	Entire population of 36 cities in the US (3 of each 3-size category x 4 regions)	1980 for population size, 1985 for pace of life, 1981 for mortality	USA
[58]	English	To model the impact of land use change, population growth and dwelling allocation on infectious disease transmission	Experimental	Cross-Sectional	Entire population of Southampton in the UK	2001-2031	Southampton, UK
[77]	English	To examine contextual factors affecting overweight and obesity among university students in China, and to examine how SES and obesity vary across geographical contexts	Ecological	Cross-Sectional	University students in China	2013	China
Søgaard, A. J., Gustad, T. K., Bjertness, E., Tell, G. S., Schei, B., Emaus, N., ... & Norwegian Epidemiological Osteoporosis Studies (NOREPOS) Research Group. (2007). Urban-rural differences in distal forearm fractures: Cohort Norway. Osteoporosis international, 18(8), 1063-1072.	English	To investigate differences in the prevalence of distal forearm fractures in areas with different degrees of urbanization	Ecological	Cross-Sectional	Norwegian Adults aged 30 and above	1994-2003	Norway
[31]	English	To present empirical observations and analytical arguments for a generalizable understanding of the consequence of urbanization on the spread of diseases	Ecological	Cross-Sectional	364 MSAs in the contiguous US	2007-2011	USA
[30]	English	To examine how the incidence of STDs changes with urban population size in US urban areas	Ecological	Cross-Sectional	364 MSAs in the contiguous US	2007-2011	USA
Marsella, A. J. (1998). Urbanization, mental health, and social deviancy: A review of issues and research. American Psychologist, 53(6), 624.	English	To review the up-to-date literature related to rural-urban differences in mental health outcomes	Review	Cross-Sectional	Publications	1998	Worldwide
[86]	English	To identify how predictive factors such as city size contribute to depression among US Vietnamese migrants	Ecological	Cross-Sectional	US Vietnamese Immigrants	2008-2012	USA
[24]	English	To estimate life expectancy at birth and mortality trends experienced by the urban workforce during the industrial revolution period.	Ecological	Longitudinal	UK & Scottish residents	1851-1901	Cities in England & Scotland
[33]	English	To investigate the relationship between urbanization and the burden of hantavirus epidemics in cities	Ecological	Longitudinal	Entire population of Hunan Province, China	1963-2010	Hunan Province, China
[38]	English	To quantify the relative contribution of three drivers of the dengue incidence in Singapore.	Ecological	Longitudinal	Singapore Residents	1974-2011	Singapore
[94]	English	To examine the current status and trends in firearm and non-firearm homicide rates by levels of urbanization	Ecological	Cross-Sectional	US teenagers ages 15-19	1979-1989	USA
[39]	Portuguese	To identify environmental and social factors associated with leishmaniasis incidence	Ecological	Cross-Sectional	23 municipalities included in the region	1980-2006	Sao Paulo, Brazil
[34]	Portuguese	To analyze the epidemiology of leprosy according to spatial distribution and living conditions in the population living in Manaus	Ecological	Cross-Sectional	Municipality of Manaus, Brazil	1998-2004	Manaus, Brazil
[89]	English	To investigate the scaling behavior of city population on the number of homicides, deaths in traffic accidents and suicides	Ecological	Cross-Sectional	Entire population of all Brazilian and US cities	1992-2009 (Brazil), 2003-2007 (US)	Brazil and USA
[78]	English	To assess the geographic distribution of obesity in the US in relation to elevation, temperature, and level of urbanization	Ecological	Cross-Sectional	US Adults	2011	USA
Van der Gulden, J. W. J., Kolk, J. J., & Verbeek, A. L. M. (1994). Socioeconomic status, urbanization grade, and prostate cancer. The Prostate, 25(2), 59-65.	English	To examine the relationship between socioeconomic status, urbanization, and prostate cancer	Ecological	Cross-Sectional	Mid-eastern Netherlands Males	1988-1990	Netherlands
[19]	English	To measure the effect of population redistribution between urban and rural areas on changes in life expectancy in Scotland between 1861 and 910	Ecological	Cross-Sectional	Population of Scotland	1861-1910	Scotland
[76]	English	To examine differences and trends in organ-specific cancer incidence according to population size	Ecological	Cross-Sectional	All registries from Gyeongsangnam-do based on Korea Central Cancer Registry (KCCR)	2008-2011	South Korea
Schram, M. E., Tedja, A. M., Spijker, R., Bos, J. D., Williams, H. C., & Spuls, P. I. (2010). Is there a rural/urban gradient in the prevalence of eczema? A systematic review. British Journal of Dermatology, 162(5), 964-973.	English	To assess the extent of an urban-rural gradient in eczema prevalence among children	Review	Cross-Sectional	Publications	2009	Worldwide
[97]	English	To compare motor vehicle crash, vehicle collision characteristics, and case fatality rates across different levels of urbanization	Ecological	Cross-Sectional	US residents ages 16 and above involved in motor vehicle crashes	1997-2010	USA
Pitel, L., Geckova, A. M., & Reijneveld, S. A. (2011). Degree of urbanization and gender differences in substance use among Slovak adolescents. International journal of public health, 56(6), 645-651.	English	To explore the association between the degree of urbanization and gender differences in smoking, binge drinking, and cannabis use among adolescents	Individual-Level	Cross-Sectional	Adolescents in 8th & 9th grade	2006	Slovakia
[87]	English	To review the current status of literature on urbanization and psychiatric disorders	Review	Cross-Sectional	Publications	1985-2010	Worldwide
[79]	English	To investigate trends in obesity prevalence in US children and adolescents by urbanization level	Ecological	Cross-Sectional	US children and adolescents ages 2-19	2001-2016	USA
[49]	English	To determine whether urbanization can explain differences in mortality rates among Hispanic children and non-Hispanic white children in US border counties	Ecological	Cross-Sectional	US children aged 1-4 years residing along US-Mexico border	2001-2015	USA
[114]	English	To examine the incidence of Perth's disease and levels of urbanization in Northern Ireland	Ecological	Cross-Sectional	Irish Children aged 0-14	1991	Northern
[40]	English	To examine tuberculosis mortality rates in 92 major US cities by population size	Ecological	Cross-Sectional	US residents with tuberculosis cause of death	1939-1943	USA
[98]	English	To examine demographic trends and mechanisms of suicide deaths within levels of urbanization in the US from 2001-2015	Ecological	Cross-Sectional	Entire population of the US	2001-2015	USA
[95]	English	To examine homicide trends by level of urbanization in US teenagers & adults aged 15-24	Ecological	Cross-Sectional	US teenagers and young adults aged 15-24 whose cause of death was homicide	1987-1995	USA
[20]	English	To examine the relationship between mortality and city size, city density and city pollution in historical (19th century) England, and in modern (2000) China	Ecological	Cross-Sectional	Population of 64 cities in the UK and 221 cities in China	1861-1890 (England), 2000 (China)	England, China
[47]	English	To examine how mortality rates from the top 5 leading causes of infant, neonatal, and post neonatal death in the United States differ by urbanization level	Ecological	Cross-Sectional	US infant deaths under age 1	2013-2015	USA
[48]	English	To examine differences in infant mortality across levels of urbanization in the US	Ecological	Cross-Sectional	US infant deaths under age 1	2014	USA
[88]	English	To examine the link between levels of urbanization and 12-month prevalence rates of psychiatric disorders in Germany	Ecological	Cross-Sectional	German adults aged 18-65	1998-1999	Germany
[46]	English	To describe variations in infant age at death in relation to urbanization and race	Ecological	Cross-Sectional	US infant fatalities	1962-1967	USA
[37]	English	To describe the relationship between urbanization and the incidence of squamous and glandular epithelium abnormalities of the cervix	Ecological	Cross-Sectional	Dutch women ages 30-60	1996-1999	Netherlands
[37]	English	To establish the baseline prevalence of genital infections and their relationship to urbanization	Ecological	Cross-Sectional	Dutch women ages 30-60	1996-1999	Netherlands
[82]	English	To examine urban-rural differences in coronary heart disease mortality among African Americans	Ecological	Cross-Sectional	African American males and females ages 35-74	1968-1986	USA
[43]	English	To examine trends in out-of-hospital cardiac arrest by level of urbanization in South Korea.	Ecological	Cross-Sectional	South Korean population with out -of-hospital cardiac arrest	2006-2010	South Korea
[45]	English	To examine variability in fertility and under 5 mortality across urban areas in West Sub-Saharan Africa	Ecological	Cross-Sectional	Urban area populations	2001-2010	West Sub-Saharan Africa
[99]	English	To examine the relationship between drug poisoning deaths and levels of urbanization	Ecological	Cross-Sectional	New Mexico population	1994-2003	USA
[26]	English	To examine mortality in New York State (Upstate NY, excluding NYC) by sex, age, and cause of death across several degrees of urbanization	Ecological	Cross-Sectional	Entire population of New York State	1949-1951	USA
[83]	English	To examine the pattern and magnitude of urban-rural variations in Coronary heart disease mortality in the US	Ecological	Longitudinal	US population ages 35 to 84	1999-2009	USA
[80]	English	To examine trends in obesity prevalence across levels of urbanization	Ecological	Cross-Sectional	US population aged 20+	2001-2006	USA
[75]	English	To examine disparities in lung cancer mortality rates among US men and women in metropolitan and non-metropolitan areas	Ecological	Cross-Sectional	US population	1950-1975	USA
Ghosn, W., Kassié, D., Jougla, E., Salem, G., Rey, G., & Rican, S. (2012). Trends in geographic mortality inequalities and their association with population changes in France, 1975–2006. The European Journal of Public Health, 23(5), 834-840.	English	To explore the ecological association between changes in cause-specific mortality inequalities and population changes across areas with different levels of urbanization	Ecological	Longitudinal	French Population under age 65	1962-2006	France
[59]	English	To examine predictable differences in influenza incidence among cities driven by population size, and to examine how these factors may affect the intensity of influenza epidemics in US cities	Experimental	Cross-Sectional	Population in 603 zip codes (in 603 cities) in the US	2002-2008	USA
Chadwick, K. A., & Collins, P. A. (2015). Examining the relationship between social support availability, urban center size, and self-perceived mental health of recent immigrants to Canada: A mixed-methods analysis. Social Science & Medicine, 128, 220-230.	English	To examine the relationship between self-perceived mental health, social support availability, and urban center size for recent immigrants to Canada	Individual-Level	Cross-Sectional	Recent Canadian immigrants	2009-2010	Canada
[28]	English	To analyze the scaling laws of several health-related variables in Brazil, Sweden, and the USA	Ecological	Cross-Sectional	Populations of cities in Brazil, Sweden, and USA	Multiple	USA, Brazil, Sweden
[41]	English	To examine racial disparities in mortality from select causes of death by degree of urbanization in the Northern and Southern US	Ecological	Cross-Sectional	US Residents	1940	USA
[36]	English	To present a model describing the efficient creation of ideas and increased productivity in cities through the formation of social ties	Experimental	Cross-Sectional	Population of 90 metropolitan areas of the USA	2008	USA and EU
[90]	English	To explore the scaling exponents for over 60 variables for the Brazilian urban system	Ecological	Cross-Sectional	Population of 5565 Brazilian municipalities	2005-2014	Brazil
[23]	English	To estimate detailed urban-rural differentials in cause-specific, age-specific, and overall death rates in the US from 1890 to 1930	Ecological	Cross-Sectional	Population of the US from 1890 to 1930	1890-1930	USA
[27]	English	To explore explanations for the rise of poor mega-cities, and how these mega-cities differ in experiencing urbanization from a historical standpoint	Ecological	Longitudinal	Urban populations	Various	100 mega-cities worldwide
[22]	English	To examine the incidence of measles and pertussis during the pre-vaccine era in US cities, and to examine the impact of urban scaling on the development of infectious disease transmission	Ecological	Cross-Sectional	US Residents diagnosed with measles or pertussis	1924-1945	USA
Cyril, S., Oldroyd, J. C., & Renzaho, A. (2013). Urbanisation, urbanicity, and health: a systematic review of the reliability and validity of urbanicity scales. BMC Public Health, 13(1), 513.	English	To assess the measurement reliability and validity of the available urbanicity scales	Review	Cross-Sectional	Publications	1970-2012	Worldwide
[71]	English	To determine the scaling relationship of death counts of four major NCDs as a function of population size. Also explores time-stability, subgroupings by size, and changes by population size	Ecological	Cross-Sectional	Population of the 395 most populous counties in the US	1999-2010	USA
[93]	English	To examine the scaling relationship of pedestrian fatality counts as a function of the population size in large US cities, and to examine the scaling relationship of non-pedestrian and total traffic fatality counts with population size	Ecological	Cross-Sectional	Population of the 116-150 largest US cities (>=150k)	1994-2011	USA
[7]	English	To examine how sociodemographic, socioeconomic, and behavioral indicators are scaling functions of city size	Ecological	Cross-Sectional	City residents in USA, China, and Germany	1990-2003	USA, China, Germany
Arbesman, S., & Christakis, N. A. (2011). Scaling of prosocial behavior in cities. Physica A: Statistical Mechanics and its Applications, 390(11), 2155-2159.	English	To examine the scaling relationship of prosocial behavior (political contributions, voting, organ donation, and census mail response) as a function of the population size	Ecological	Cross-Sectional	Population of US CBSAs	Various	USA
[32]	English	To examine the evolution and current status of the AIDS epidemic in Brazil using growth patterns and scaling laws	Ecological	Cross-Sectional	Population of Brazilian municipalities	1980 to 2012 (2000 and 2010 for the scaling analysis)	Brazil
[21]	English	To examine the relationship between annual pneumonia and influenza mortality rates in 2 time periods (pre/post pandemic), and the scaling of mortality with population size	Ecological	Cross-Sectional	Population of 66 US cities above 100k pop in 1920	1910-1917 (pre), 1918-1920 (post)	USA
Takano, T., Fu, J., Nakamura, K., Uji, K., Fukuda, Y., Watanabe, M., & Nakajima, H. (2002). Age-adjusted mortality and its association to variations in urban conditions in Shanghai. Health Policy, 61(3), 239-253.	English	To explore the association between mortality and urbanization in Shanghai	Ecological	Cross-Sectional	Shanghai Residents	1995-1997	China
Session, T. F. (1975). REGIONAL COMMITTEE FOR THE EASTERN MEDITERRANEAN.	English	To examine the association between urbanization and childhood behavioral problems	Individual-Level	Cross-Sectional	Sudanese Children ages 3-15	1980	Sudan
[84]	English	To examine regional and urbanization differentials in CHD mortality among White male adults	Ecological	Longitudinal	White Males ages 35-74	1968-1985	USA
Gomez-Lievano, A., Patterson-Lomba, O., & Hausmann, R. (2017). Explaining the prevalence, scaling and variance of urban phenomena. Nature Human Behaviour, 1(1), 0012.	English	To examine the urban scaling of several urban phenomena including sexually transmitted infections	Ecological	Cross-Sectional	US cases	2007-2011	USA
[91]	English	To investigate the relationships between crime and urban metrics	Ecological	Cross-Sectional	Population of Brazilian municipalities	2000	Brazil
[12]	English	To compare cities relative to its peers in terms of population	Ecological	Cross-Sectional	US population living in metropolitan areas	1969-2006	USA
[50]	English	To quantify geographical patterns during the autumn and winter waves of the 1918 flu pandemic in English and Welsh cities	Ecological	Cross-Sectional	Population of England and Wales (247 towns/cities, 58 rural areas)	1918-1919	England and Wales
[92]	English	To establish general properties of the statistics of urban indicators (using crime as an example) in the limit of high granularity and to investigate if and how urban scaling laws emerge and are related to Zipf's law for the population size of cities	Ecological	Cross-Sectional	Population of Brazilian, Colombian, and Mexican cities	2003-2009	Brazil, Colombia, Mexico
[13]	English	To investigate the universality and robustness of scaling laws for urban systems in Brazil	Ecological	Cross-Sectional	Population of Brazilian metropolitan areas and municipalities	2010	Brazil
[51]	English	To study the hypothesis that differences in mortality during the 1918 influenza pandemic in US cities resulted from the wide variety of public health measures	Ecological	Cross-Sectional	45 US cities	1918-1919	USA
Bjørnstad, O. N., Finkenstädt, B. F., & Grenfell, B. T. (2002). Dynamics of measles epidemics: estimating scaling of transmission rates using a time series SIR model. *Ecological monographs*, *72*(2), 169-184.	English	To develop a mechanistic model of measles dynamics to understand endemic dynamics	Experimental	Cross-Sectional	60 cities in England and Wales	1944-1966	England and Wales
[54]	English	To explore the impact of rurality on the 1918 influenza pandemic in New Zealand	Ecological	Cross-Sectional	4 cities, 111 towns, and 97 counties of New Zealand	1918	New Zealand
[67]	English	To examine spatial heterogeneity in transmission probability by population size	Ecological	Cross-Sectional	845 cities and 457 rural districts, and 60 largest towns and cities (post vaccination period)	1950-1967 and 1972-1980 (just rural-urban comparison)	England and Wales
[60]	English	To characterize the pattern of local measles epidemics in terms of a balance of local factors (birth rate and population size) and regional factors (coupling)	Ecological	Cross-Sectional	60 cities in England and Wales	1944-1966	England and Wales
[62]	English	To explore the relationship between the size of a community and the mean period between epidemics, and to explore the existence of a critical community size above which fade out of infections was unlikely	Ecological	Cross-Sectional	19 cities in England and Wales	1940-1956	England and Wales
[63]	English	To explore the critical community size for continuous measles transmission in US cities	Ecological	Cross-Sectional	24 cities in the US and Canada	1921-1940	USA and Canada
[68]	English	To develop a more realistic mechanistic model of measles epidemics that fits better the critical community size	Experimental	Cross-Sectional	60 towns in England & Wales	1944-1968	England and Wales
[65]	English	To develop a model to understand the interaction between community size and birth rate on measles fadeouts	Experimental	Cross-Sectional	60 towns in England & Wales	1944-1968	England and Wales
[69]	English	To model non-stationarity and spatial heterogeneities in recurrent epidemics of measles	Experimental	Cross-Sectional	354 Administrative Areas in England & Wales	1944-1994	England and Wales
[64]	English	To evaluate the effect of vaccination on outbreak dynamics using a metapopulation model consisting of communities of different sizes	Experimental	Cross-Sectional	40 Communities in sub-Saharan Africa	1986-2005	Niger
[55]	English	To model influenza spread between US cities across 8 influenza seasons using population size as a proxy for location susceptibility	Experimental	Cross-Sectional	310 US Locations	2002-2010	USA
[70]	English	To examine the effect of population size on measles endemicity and the evolutionary implications of population size and measles cases in humans, using insular data	Ecological	Cross-Sectional	19 Islands around the world	1949-1964	Several
Salje, H., Lessler, J., Berry, I. M., Melendrez, M. C., Endy, T., Kalayanarooj, S., ... & Thaisomboonsuk, B. (2017). Dengue diversity across spatial and temporal scales: Local structure and the effect of host population size. Science, 355(6331), 1302-1306.	English	To examine the role of local population size in dictating the number of transmission chains	Individual-Level	Cross-Sectional	Hospitals in Thailand	1994-2010	Several
[56]	English	To model the spatial transmission of influenza using population size as a proxy for location susceptibility	Experimental	Cross-Sectional	271 US cities & suburban areas	2009	USA
[52]	English	To better characterize the spread of the 1918 pandemic influenza between cities	Ecological	Cross-Sectional	246 population centers in England, Wales, and the US	1918-1919	USA, England, Wales
[57]	English	To analyze the spatial dynamics of interpandemic influenza epidemics between 1972 and 2002 using data for the 49 contiguous US states	Experimental	Cross-Sectional	48 continental US States & the District of Columbia	1972-2002	USA
[61]	English	To understand the network that governs regional spread of measles and the consequences on local epidemics	Experimental	Cross-Sectional	954 urban locations in England and Wales	1944-1967	England and Wales
[53]	English	To estimate the reproductive number of 1918 pandemic influenza	Experimental	Cross-Sectional	45 US Cities	1918	USA
Study Setting Country	Country Income	City Definition	Exposure	Outcome	Outcome Measure	WHO Class	Results & Conclusion
USA	High	Official Metropolitan Area	Population Size & Relative Location	Prevalent cases of HIV	Race-specific HIV prevalence rate ratios	CMNN	Racial/ethnic disparities were observed for all levels of urbanization. HIV prevalence increased with level of urbanization among Whites and Blacks. After controlling for poverty in large urban counties, there were no significant racial/ethnic disparities. In non-urban counties, racial/ethnic disparities existed after adjusting for poverty. The association between HIV prevalence and poverty varies by level of urbanization.
USA	High	Administrative Unit	Categorical Population Size	Coronary heart disease mortality	Average Annual CHD death rate per 1000	MCM	In the first period, mortality in urban residents increases with urbanization, while in the second period it is stable or decreases.
Several	High	Other: Researcher Defined	Categorical Population Size	Smoking	Prevalence rates, Odds Ratio	OTHER	In all countries, smoking prevalence was higher in urban areas. Smoking prevalence was directly related to level of urbanization. There were no significant differences in annual rate of change in smoking prevalence between urban and rural areas.
USA	High	Official Metropolitan Area	Population Size & Relative Location	Physical inactivity	Point Prevalence, Odds Ratio	OTHER	The prevalence of physical inactivity was highest in rural areas, and lowest in metropolitan and large urban areas of the US. When compared to the western US, the highest likelihood of physical inactivity was highest in the southern region across all levels of urbanization with rural areas having the highest odds of physical inactivity.
Brazil	Low	Administrative Unit	Urbanization	Asthma hospital admissions, Asthma mortality	Odds Ratio	NCD	Municipalities with higher proportions of urban population had higher odds of high asthma hospital admissions and asthma deaths. Increasing urban populations over time was associated with lower odds of reducing asthma hospital admissions and asthma deaths.
USA	High	Official Metropolitan Area	Population Size & Relative Location	Sepsis mortality	Age-adjusted mortality rates	MCM	Sepsis associated mortality rates were higher in Blacks than Whites across all levels of urbanization. For both Blacks & Whites, sepsis mortality rates were highest in micropolitan areas and lowest in fringe metropolitan areas.
Italy	High	Official Metropolitan Area	Categorical Population Size	Perceived Insecurity	Perceived Insecurity Questionnaire Scores	OTHER	Perceived Insecurity was more frequent in big cities with a population with populations of over 300,000.
USA	High	Administrative Unit	Continuous Population Size	Lung Cancer Mortality	Population-weighted mortality rates	MCM	Higher rates of mortality were found in more urbanized counties. The urban-rural gradient was significant for both males and females during each of the study periods.
Japan	High	Administrative Unit	Categorical Population Size	Hysteria or Endogenous Depression	Proportion of hysteria or depression cases	NCD	The proportion of hysteria is lower than the proportion of depression in all population sizes except in cities.
Several	Low	Administrative Unit	Population Growth	HIV incidence	Incidence Rate	CMNN	City growth was not concurrent with the emergence and spread of HIV, although in central African cities it seems to have originated in the biggest cities.
USA	High	Administrative Unit	Population Size & Relative Location	Acute lymphocytic leukemia, non-Hodgkin lymphoma, Acute nonlymphocytic leukemia	Incidence rate	NCD	Among White children of both sexes, incidence of acute lymphocytic leukemia was lower in rural areas. There were no urban-rural gradients for non-Hodgkin lymphoma and Acute nonlymphocytic leukemia.
Mexico	Low	Administrative Unit	Categorical Population Size	Mortality due to birth defect (MBD) among children younger than 5 year of age	Among cities above P80 of MBD, MBD was categorized in deciles, and cities in D89-80 were considered high priority cities; D90+ very high priority; under D80 were classified as other priority	MCM	Municipalities with high and very priority in MBD are highly urbanized, and concentrate the majority of the production units and GDP in industry and transportation.
Brazil	Low	Administrative Unit	Categorical Population Size	Hospitalizations due to a set of conditions ranging from vaccine preventable diseases to hypertension	Hospitalization counts for each municipality	CMNN, NCD, INJ	There's an inverted U shape, with medium sized municipalities having the highest rates, followed by both small and medium-large municipalities, and finally the largest municipalities shaving the lowest rates by far.
USA	High	Official Metropolitan Area	Categorical Population Size	Coronary heart disease (CHD) mortality	Age adjusted CHD mortality per 100,000	MCM	No association between size-region and CHD at the city level.
England	High	Administrative Unit	Population Growth	Influenza Transmission	Number of infected people	CMNN	The simulation model not accounting for age structure suggests a small effect of population growth on influenza transmission. When considering age structure, the simulated iterations suggest that flu infection marginally increases with population growth, but overall decreases with growth and time.
China	Low	Administrative Unit	Population Size & Relative Location	Overweight, Obesity	Point Prevalence & Odds Ratios	NCD	There was a higher prevalence of obesity in urban areas than rural areas, independent of individual factors. Students from larger cities were more likely to be obese then students from smaller cities. Larger and wealthier cities attenuates the positive association between SES and obesity.
Norway	High	Administrative Unit	Continuous Population Size	Self-reported cases of distal forearm fracture	Prevalence rates, Odds Ratio	INJ	The prevalence of forearm fractures increased with increasing degree of urbanization for both genders
USA	High	Official Metropolitan Area	Population Size & Relative Location	Incidence of chlamydia, gonorrhea, and syphilis	Number of incident cases	CMNN	All three diseases show superlinear scaling. Socioeconomic covariates that increase prevalence reduce the scaling coefficient. For example, poorer larger MSAs don’t have such a high prevalence, as compared to wealthier MSAs.STDs with higher scaling exponents also have lower intercepts and variance given population size (and vice-versa).
USA	High	Official Metropolitan Area	Population Size & Relative Location	Incidence of chlamydia, gonorrhea, and syphilis	Number of incident cases	CMNN	After controlling for several socioeconomic factors, a superlinear relation between STD incidence and urban population size exists. Also, the percentage of African Americans, education, income, and income inequalities were found to have a sig. impact on STD incidence.
Several	NA	Other: Researcher Defined	Urbanization	Mental Health and Social Deviancy	Several	NCD, OTHER	Several
USA	High	Administrative Unit	Continuous Population Size	Depressive symptoms	Prevalence Rates	NCD	Depression prevalence was higher in large cities than in medium sized cities.
Several	High	Other: Unclear	Categorical Population Size	Life Expectancy (LE)	LE in years	ACM	There are apparent overall trends in life expectancy at birth for each decade of the 19th century, which accompanied rapid population growth in the largest cities. In England, aside from the southern towns, all the other cities display life expectancies below the national average, especially during the mid-century period. Bigger cities tend to have lower life expectancy.
China	Low	Administrative Unit	Population Growth	Hantavirus induced hemorrhagic fever with renal syndrome (HFRS)	HFRS Incidence per 10,000	CMNN	U shape between size and incidence over time within cities (so peak at mid urbanization). Migration also prolongs epidemics. All seems to be connected through economic growth.
Singapore	High	Administrative Unit	Continuous Population Size	Dengue	Incidence	CMNN	Population growth was the leading independent factor associated with the increase in dengue cases observed in Singapore over the past 40 years.
USA	High	Official Metropolitan Area	Population Size & Relative Location	Homicide	Prevalence Rates	INJ	Firearm homicide rates were higher in metropolitan counties than non-metropolitan counties. Within metropolitan counties, homicide rates were higher in core counties compared to other levels of urbanization. These differences were smaller in non-firearm homicide rates.
Brazil	Low	Administrative Unit	Continuous Population Size	American cutaneous leishmaniasis (ACL)	Incidence Rate	CMNN	Leishmaniasis was positively correlated with urbanization. Higher incidence of ACL was associated with higher level of urbanization (during 1998-200); and mean urban population size (fduring2001-2003 and 2004-2006).
Brazil	Low	Administrative Unit	Urbanization	Endemic disease distribution	Lprosy detection rate: hyper-endemic (>= 4 per 10,000 inhabitant); very high (4 to 2), high (2 to 1); medium (1 to 0.2), low (<0.2)	CMNN	The chances of leprosy cases in a certain census tract increase in proportion to the number of cases in children under 15 and to the worsening of living conditions of the population living in Manaus.
Several	NA	Official Metropolitan Area & Administrative Unit	Continuous Population Size	Suicide, Homicide, Traffic Accident mortality	Number of homicides, deaths in traffic accidents, and suicides	INJ	Homicides show superlinear scaling, traffic accidents linear scaling, and suicides sublinear scaling.
USA	High	Official Metropolitan Area	Population Size & Relative Location	Obesity	Point Prevalence	NCD	Compared to large metropolitan counties, non-metropolitan/rural counties had the highest prevalence of obesity followed by small and medium sized metropolitan counties.
Netherlands	High	Administrative Unit	Categorical Population Size	Malign Prostate Tumor	Incidence, Rate Ratio	NCD	Slight non-significant trend of higher risk for men living in rural areas, suggests no significant relationship between prostate cancer incidence and urbanization.
Scotland	High	Administrative Unit	Categorical Population Size	Life Expectancy at birth, Mortality Rates	Life Expectancy in years, Mortality Rate	ACM	Results suggest higher mortality in urban areas, and an urbanization penalty accompanying population redistribution from rural to urban areas, indicating that rural to urban migration its associated population change has a negative effect on life expectancy.
South Korea	High	Administrative Unit	Categorical Population Size	Organ-specific cancer	Incidence Rate	NCD	Thyroid & Colorectal cancer incidence was much lower in rural areas than in urban areas. Gastric & Lung cancer incidence was more common in rural areas. Thyroid cancer incidence higher in metropolitan vs. non-metropolitan areas.
Several	NA	Other: Researcher Defined	Categorical Population Size	Eczema	Relative Risk, Prevalence Rates	NCD	The prevalence of eczema was higher in urban areas. The relative risk of eczema was significantly higher in urban areas.
USA	High	Official Metropolitan Area	Categorical Population Size	Motor vehicle crash mortality	Odds Ratio	INJ	Occupants of vehicles crashing in rural areas and small cities experience a higher likelihood of dying than those in central cities and suburban cities.
Slovakia	High	Administrative Unit	Categorical Population Size	Smoking habit, cannabis, and alcohol consumption	Self-reported Prevalence Rates	NCD	In females, lower degree of urbanization is associated with significant lower consumption (all 3 substances), while the prevalence remained constant in males.
Several	NA	Other: Researcher Defined	Urbanization	Psychiatric disorders	Prevalence Rates	NCD	Prevalence rates for psychiatric disorders were higher in urban areas compared to rural areas. Mood and anxiety disorders were higher in urban areas, while rates for substance use disorders did not show a difference.
USA	High	Official Metropolitan Area	Population Size & Relative Location	Obesity and Severe Obesity	Prevalence Rates	NCD	From 2001-2016 there is a linear trend in obesity & severe obesity prevalence across levels of urbanization. There are patterns in BMI distribution by urbanization. No significant difference in obesity across levels of urbanization. Severe obesity was significantly higher in non-MSAs than Large-MSAs.
USA	High	Official Metropolitan Area	Population Size & Relative Location	Child mortality	Mortality Rates	MCM	Mortality rates in border Hispanic children is highest compared to the other groups. Mortality rates increased with declining urbanization for all groups. Among US children in border counties, there is a significant negative time trend in mortality rates for large central and large fringe areas.
Northern Ireland	High	Administrative Unit	Categorical Population Size	Perthes' disease	Prevalence Rates	NCD	There was no evidence of an increased risk of Perthes disease in urban areas.
USA	High	Administrative Unit	Categorical Population Size	Tuberculosis mortality	Mortality Rates	MCM	Tuberculosis mortality rate is higher with increasing population size and is greater among Non-Whites. Rates for the 1939-1941 period are higher than those in the 1942-1943 period.
USA	High	Administrative Unit	Population Size & Relative Location	Suicide mortality	Suicide age-adjusted mortality per 100,000	INJ	Suicide mortality rate is higher in non-metropolitan counties, followed by medium-small metro, and large. Differences are widening. Especially strong among males, midlife, Non-Blacks, and by firearms.
USA	High	Official Metropolitan Area	Population Size & Relative Location	Homicide mortality	Mortality Rates, Annual Percent Change	INJ	Homicide rates began to decline between 1993-1995 across all levels of urbanization. In Large & Fringe MSAs firearm homicide increased from 1987-1992 and then decreased. Mortality rates are higher in Black males compared to White males (similar pattern for women, smaller in magnitude). There is a gradient of increasing homicide mortality rate with increasing urbanization.
Several	NA	Administrative Unit	Continuous Population Size	Age-adjusted mortality	Mortality rates	ACM	In 19th century England, bigger cities had higher mortality; in Modern China bigger cities have lower mortality.
USA	High	Official Metropolitan Area	Population Size & Relative Location	5 Leading Causes of Infant Death (Congenital Malformations, Low Birth Weight, SIDS, Maternal Complications, Unintentional Injuries)	Mortality Rates	MCM	Infant death rates were higher in rural counties than in large urban counties. Post neonatal mortality rates for SIDS and congenital malformation and unintentional injuries were highest in rural areas and lowest in large urban areas.
USA	High	Official Metropolitan Area	Population Size & Relative Location	Infant mortality	Mortality Rates	ACM	Infant mortality rates decreased as urbanization level increased for neonatal and post-neonatal deaths. Among Hispanic mothers, IMR was higher in small and medium urban counties compared with large urban counties. Infant mortality rates for rural counties were similar to the rate for small and medium urban counties.
Germany	High	Administrative Unit	Categorical Population Size	Psychiatric disorders	Prevalence Rates	NCD	Higher levels of urbanization were linked to higher 12-month prevalence rates for all major psychiatric disorders except substance abuse and psychotic disorders. Weighted prevalence of all disorders were highest in urbanized areas.
USA	High	Official Metropolitan Area	Population Size & Relative Location	Perinatal & Infant mortality	Mortality Rates	ACM	Mortality rates in infants older than 7 days old increase progressively as degree of urbanization decreases, this relationship is stronger during the post-neonatal period. The relative disadvantage of non-white infant mortality increases with both age and decreasing urbanization. After one day of life, infant mortality increases as degree of urbanization decreases in greater MSA compared to rural areas.
Netherlands	High	Official Metropolitan Area	Categorical Population Size	Squamous & Glandular epithelium abnormalities of the cervix	Incidence Rate	NCD	The incidence of squamous and glandular abnormalities were highest in women who lived in large cities.
Netherlands	High	Official Metropolitan Area	Categorical Population Size	HPV, Trichomonas, Candida, Gardnerella, Actinomyces, and Chlamydia	Prevalence Rates	CMNN	Higher prevalence of HPV, bacterial vaginosis, and trichomonas was present in more urbanized areas, but Candida was not.
USA	High	Official Metropolitan Area	Population Size & Relative Location	Coronary heart disease mortality	Mortality Rates	MCM	African American males in greater metropolitan areas had 29% excess coronary heart disease mortality compared to isolated rural areas. While African American women had 45% excess mortality. There was an urban rural gradient in coronary heart disease mortality. Women experienced greater relative declines in mortality and smaller absolute declines than males.
South Korea	High	Administrative Unit	Categorical Population Size	Out-of-hospital cardiac arrest	Survival-to-admission Rate, Survival-to-discharge Rate, Incident Rates	CMNN, NCD, INJ	The standardized incidence rate and survival to discharge rate of EMS-assessed OHCAs increased annually in metropolitan and urban communities but did not increase in rural communities.
Several	Low	Other: Researcher Defined	Categorical Population Size	Fertility and under 5 mortality	Total fertility rates and under-5 survival rates	ACM	Fertility gradient with lower total fertility rates and under 5 survival rates in big urban areas. Under 5 survival is higher in larger cities (similar for all >150K) compared to cities <150K.
USA	High	Official Metropolitan Area	Population Size & Relative Location	Drug poisoning deaths due to illicit (cocaine, heroin), prescription (opioids, non-methadone painkillers, methadone) and over-the-counter drugs (alcohol)	Mortality rates	MCM	Metropolitan areas had the highest rates of all drug-poisoning death, any illicit drug, heroin, and cocaine, methadone, and over-the-counter drugs. Nonstatistical areas had the highest rates of opioid painkillers other than methadone. Micropolitan areas had the highest rate of alcohol and drug cointoxication.
USA	High	Official Metropolitan Area	Categorical Population Size	Mortality, several causes	Mortality per 1,000, age-adjusted and age-specific	ACM	The overall mortality rate was highest in the central cities, and the total mortality rates for both urban and rural areas outside these cities were higher in nonmetro than metro areas. By age, there was lower mortality among young people and marked excess mortality between ages 35-65 in cities, the difference being greater for males than for females. In general, the mortality from every cause of death, except accidents and suicides follow the same pattern as all-cause mortality with the highest rates in central cities and the lowest in rural areas. The greatest deviation in mortality patterns in central cities is contributable to TB, liver cirrhosis, and Syphilis. Closely followed by arteriosclerotic heart disease (including CHD).
USA	High	Official Metropolitan Area	Population Size & Relative Location	Coronary heart disease mortality	Age-adjusted mortality rates	MCM	Age-adjusted CHD mortality declined over time for the population in all three categories of urbanization, but declines were greater in urban than in rural areas.
USA	High	Official Metropolitan Area	Population Size & Relative Location	Obesity, Severe Obesity	Age-Adjusted Prevalence Rates	NCD	There was a significantly increasing linear trend in obesity prevalence from large MSAs to non-MSAs. Individuals living in medium/small MSAs had higher age adjusted prevalence of obesity and severe obesity compared to those living in large MSAs.
USA	High	Official Metropolitan Area	Population Size & Relative Location	Site-specific cancer mortality	Age-adjusted mortality rates	MCM	Cancer mortality increased with urbanization level but the differences between the most and the least urban categories declined over time.
France	High	Administrative Unit	Population Growth	All-cause mortality	Age-adjusted mortality rates	ACM	Premature mortality declined in urban cores that had large increases in population between 1962 and 1990. Premature mortality also decreased in peri-urban areas with different profiles of population dynamics (increase/decrease), but in rural area mortality increased.
USA	High	Administrative Unit	Continuous Population Size	Influenza-like illness	Epidemic intensity of influenza	CMNN	Epidemic intensity is higher in smaller cities because of higher base transmission potential in larger cities leading to diffuse off-peak epidemics that create herd immunity.
Canada	High	Official Metropolitan Area	Categorical Population Size	Self-perceived mental health	Count, Odds Ratio	NCD	Recent immigrants in small urban areas are twice as likely to report low self-perceived mental health compared to those living in large urban centers.
Several	NA	Administrative Unit	Continuous Population Size	Noncommunicable & Infectious diseases, external causes of death, behaviors, healthcare availability	Prevalence or Incidence or mortality counts	CMNN, MCM	There's a diversity of results here. In general IDs that go person-to-person scale superlinearly except those that relate to resource-poor settings. Crimes scale superlinearly. Metabolic causes are sublinear or linear. Risk factors scale sublinearly (but maybe not for Brazil). Health resources scale superlinearly Results indicates that using rates as indicators to compare cities with different population sizes may be insufficient.
USA	High	Administrative Unit	Categorical Population Size	Cause-specific mortality (Tuberculosis, Influenza, Nephritis, Pneumonia, Cardiovascular disease, Syphilis, Homicide)	Mortality Rates	MCM	In the southern US, Non-White cause specific rural mortality rates were lowest. In the northern US, the cause specific rural mortality rates were higher than those in larger cities.
Several	High	Official Metropolitan Area	Continuous Population Size	AIDS/HIV incidence	New AIDS/HIV Cases per square mile	CMNN	Increases in density and proximity of populations in cities leads to super-linear growth of social tie density for urban populations. Additionally, the diffusion rate along social ties accurately reproduces the empirically measures scaling of features such as AIDS/HIV infections, communication, and GDP.
Brazil	Low	Administrative Unit	Continuous Population Size	Deaths by external causes (traffic accidents, suicides, homicides)	Counts	INJ	Traffic accidents and homicides scaled superlinearly, suicides sublinearly.
USA	High	Administrative Unit	Categorical Population Size	Age-adjusted mortality, age-specific mortality, cause-specific mortality	Mortality rates per 100,000	MCM	There is a shift from higher ID mortality in cities (and more the larger the city is) to NCDs with similar mortality across the urban spectrum. Overall, mortality was <1918 higher in bigger cities than in smaller cities than in rural areas. In 1918 highest mortality was in smaller cities and decreased as cities grew in size (and even lower in rural areas). From there on, the same pattern persisted.
Several	NA	Administrative Unit	Population Growth	Unclear	Unclear	ACM	Urban wages display an inverted U-shape with respect to city population size.
USA	High	Administrative Unit	Continuous Population Size	Cryptic incidence of measles and pertussis	Cryptic Incidence Rates	CMNN	Cryptic incidence is concentrated. Pertussis, can sustain low but non-zero incidence in much smaller populations than measles owing to a longer infectious period and lower transmission rate.
Several	NA	Other: Unclear	Urbanization	Several health outcomes	Several	CMNN, NCD, INJ, OTHER	Increased urbanization was associated with deleterious outcomes. Urbanicity measures differed across studies.
USA	High	Administrative Unit	Continuous Population Size	Mortality by cancer, CVD, endocrine/metabolic, respiratory	Disease-specific mortality counts	MCM	All diseases show superlinear scaling. When restricting to the biggest counties, they show sublinearity (esp. cancer cvd and respiratory). These scaling relationships are time-invariant. These scaling relationships are not explained by other covariates.
USA	High	Other: Researcher Defined	Continuous Population Size	Pedestrian fatalities, non-pedestrian fatalities, total traffic fatalities	Traffic death counts	INJ	Pedestrian deaths is linear, non-pedestrian deaths is superlinear. Time invariant. Pedestrian deaths are strongly sublinear in the largest cities. Same for total deaths., and even non-pedestrian (in the case of the largest cities). So, the larger the city threshold, the more sublinear the relationship is.
Several	High	Official Metropolitan Area & Administrative Unit	Continuous Population Size	New AIDS cases, Serious Crimes	Counts	CMNN	New cases of AIDS and serious crimes follow a superlinear scaling law
USA	High	Official Metropolitan Area	Population Size & Relative Location	Prosocial behavior	Number of political contributions and total dollar amounts, total number of votes, number of organs donated, responses to the census	OTHER	Organ donation scales linearly.
Brazil	Low	Administrative Unit	Continuous Population Size	New AIDS cases	Number of new AIDS cases	CMNN	Strong superlinear scaling law for AIDS.
USA	High	Administrative Unit	Continuous Population Size	Influenza and pneumonia mortality	Influenza and pneumonia death counts	MCM	Pneumonia death counts had a linear relationship, but influenza counts followed a strongly sublinear relationship in 1918. It was linear after the pandemic or before.
China	Low	Administrative Unit	Population Growth	Age-adjusted mortality	Mortality Rates	ACM	Higher population density and per capita floor-space significantly positive and negatively associated with mortality rates, respectively.
Sudan	Low	Administrative Unit	Population Growth	Child behavioral problems	Prevalence Rates	OTHER	There were no significant differences in the prevalence of child behavior problems among comparison groups.
USA	High	Official Metropolitan Area	Population Size & Relative Location	Coronary heart disease mortality	Mortality Rate	MCM	Coronary heart disease mortality declined across all region-urbanization groups. The core metro area had the lowest mortality rates in the South, but the highest in the other regions.
USA	High	Official Metropolitan Area	Continuous Population Size	Chlamydia and Syphilis	5-year cumulative incidence rate	CMNN	Both Chlamydia and Syphilis had superlinear scaling behavior.
Brazil	Low	Administrative Unit	Continuous Population Size	Homicides	Count	INJ	Superlinear scaling of homicides.
USA	High	Official Metropolitan Area	Continuous Population Size	Homicides	Number of homicides	INJ	Superlinear scaling of homicides, and homicides are weakly correlated with GDP/income after accounting for scaling.
Several	High	Administrative Unit	Continuous Population Size	Transmissibility, mortality, and timing of the autumn and winter pandemic influenza waves	R0 for transmissibility and mortality counts and rates	CMNN	No statistically significant association between size and transmissibility, much higher mortality in urban than rural areas, with sublinearity in rural areas and linearity in urban areas, and EARLIER pandemic onset in areas with larger population.
Several	NA	Official Metropolitan Area	Continuous Population Size	Homicides	Number of homicides	INJ	Superlinear scaling of homicides.
Brazil	Low	Official Metropolitan Area & Administrative Unit	Continuous Population Size	Homicides	Number of homicides	INJ	Superlinear scaling of homicides in all cities, but if restricted to at least 10 homicides there's sublinear scaling.
USA	High	Administrative Unit	Population Size	Excess mortality	Excess mortality during the autumn wave of the 1918 influenza pandemic	MCM	Population size not associated with total or peak mortality.
Several	High	Other	Population Size	Measles incidence	Mean weekly biweekly case count and proportion biweekly periods with 0 counts	CMNN	Large cities have regular endemic disease cycles with no fadeouts at all (>300k pop), medium sized cities have occasional brief fadeouts, and smaller cities have long fadeouts with irregular outbreaks. There is no relationship between city size and R0, but transmission rates are higher with bigger cities (frequency dependent transmission).
New Zealand	High	Other	Population Size	Influenza mortality rate	Mortality rate per 1000 per 3 months	MCM	Mortality was higher in urban than in rural areas, but within them it was highest in small towns, followed by large towns, and by cities (lowest mortality among urban areas).
Several	High	Other	Population Size	Measles incidence	Measles case count	CMNN	Measles epidemics fadeout frequency and duration decreases with city size, so that in the smallest areas fadeouts are long and frequent, while as size increases they become shorter and more frequent, with big cities having no fadeouts. Epidemics start in larger cities and then move to smaller ones and rural areas.
Several	High	Other	Population Size	Measles incidence	Mean weekly biweekly case count and proportion biweekly periods with 0 counts	CMNN	Measles scales linearly with city size overall but differs by type of epidemic: in main epidemic year, the scaling is slightly superlinear (1.04) while in minor epidemic years it is strongly sublinear (0.74). The probability of fadeouts is much less common in bigger cities. Local deterministic dynamics (size) predominate during major epidemics, while they are less important during minor epidemics and fadeouts.
Several	High	Other	Population Size	Measles incidence	Measles fadeouts probability	CMNN	Cities above 200-250k people do not show fadeouts, while in cities smaller than that, the probability of fadeout decreases with size.
Several	High	Other	Population Size	Measles incidence	Measles fadeouts probability	CMNN	Cities above 250-300k people do not experience fadeouts in measles incidence.
Several	High	Other	Population Size	Measles incidence	Measles fadeouts probability	CMNN	Smaller populations (150K) experience longer total fadeout durations and a higher number of fadeouts per year.
Several	High	Other	Population Size	Measles Incidence	Annual Measles fadeouts	CMNN	Higher birth rates lower the critical community size, and in these settings vaccination increases the critical community size.
Several	High	Administrative Unit	Population Size	Measles incidence	Measles Cases	CMNN	There are spatial heterogeneities in measles epidemics: epidemics travel from large cities to smaller towns, specifically going from large core cities to satellite towns of these cities.
Niger	Low	Administrative Unit	Population Size	Measles Cases	Proportion of weeks with 0 cases	CMNN	Larger cities have lower proportions of weeks with 0 cases of measles. However, critical community size was an order of magnitude larger than for UK/US cities.
USA	High	Other	Population Size	Influenza-like-illness Incidence	Incidence	CMNN	More populated locations are at highest risk of influenza transmission; density doesn’t matter. However, this was weak: local spread (distance-based) predominated over hierarchical spread (larger to smaller cities). In fact, size only marginally affected a city's risk of obtaining influenza early in the pandemic. This may be related to a younger (more mobile) population in larger cities: confounding. SECONDARY ANALYSIS important: while at the state level size is important to determine synchrony, it looks like at the city level geographic distance predominates (EXAMPLE OF MAUP).
Several	NA	Other	Population Size	Measles Cases	Count and % months with measles	CMNN	Larger populations in insular communities results in prolonged endemicity of measles. Moreover, higher density prolongs epidemics in smaller islands.
Several	Upper-Mid	Other	Population Size	Dengue Transmission	Number of transmission chains	CMNN	The number of effective transmission chains increases with population size, indicating relatively higher risk of dengue transmission in larger populations. However, this tapers off at higher levels of density.
USA	High	Administrative Unit	Population Size	Influenza-like-illness Incidence	Pandemic onset timings	CMNN	In 2009 H1N1 pandemic locations with large populations are at higher risk of infleunza transmission, but this association is weak, and transmission shows a spatial component starting in the Southeastern US.
Several	High	Other	Population Size	Influenza Transmission	Transmission proxied by Influenza and pneumonia mortality	CMNN	As population size increases, the susceptibility of the city increases but more slowly (sublinearly). Population size of infectious city (origin) is very weakly associated (consistent with 95; opposed to Measles, where it matters). This indicates weaker spatial hierarchies than measles (e.g., from large to smaller cities).
USA	High	Other	Population Size	Influenza Mortality	Weekly excess mortality rates from pneumonia and influenza	CMNN	Bigger states have synchronized epidemics, while smaller states have erratic behavior. Size of the state is not associated with transmission.
Several	High	Other	Population Size	Measles Transmission	Measles cases and fadeouts	CMNN	New results from this paper: larger cities emit relatively more infections than smaller cities; this is also spatially patterned (larger cities have surrounding smaller cities). parameter of "transfer of infection based on donor population" is superlinear. However, city size of recipient city does not influence transmission (R0 is constant; consistent with previous papers showing linearity of measles).
USA	High	Other	Population Size	Influenza Transmission	Influenza Reproductive Number	CMNN	Influenza transmission was weakly correlated with city size.
